# Hemoglobin S and C affect protein export in *Plasmodium falciparum*-infected erythrocytes

**DOI:** 10.1242/bio.201410942

**Published:** 2015-02-20

**Authors:** Nicole Kilian, Sirikamol Srismith, Martin Dittmer, Djeneba Ouermi, Cyrille Bisseye, Jacques Simpore, Marek Cyrklaff, Cecilia P. Sanchez, Michael Lanzer

**Affiliations:** 1 Center of Infectious Diseases, Parasitology, Heidelberg University, Im Neuenheimer Feld 324, 69120 Heidelberg, Germany; 2 Biomolecular Research Center Pietro Annigoni, University of Ouagadougou, 01 BP 364 Ouagadougou, Burkina Faso

**Keywords:** Hemoglobinopathy, Protein export, Malaria, *P. falciparum*

## Abstract

Malaria is a potentially deadly disease. However, not every infected person develops severe symptoms. Some people are protected by naturally occurring mechanisms that frequently involve inheritable modifications in their hemoglobin. The best studied protective hemoglobins are the sickle cell hemoglobin (HbS) and hemoglobin C (HbC) which both result from a single amino acid substitution in β-globin: glutamic acid at position 6 is replaced by valine or lysine, respectively. How these hemoglobinopathies protect from severe malaria is only partly understood. Models currently proposed in the literature include reduced disease-mediating cytoadherence of parasitized hemoglobinopathic erythrocytes, impaired intraerythrocytic development of the parasite, dampened inflammatory responses, or a combination thereof. Using a conditional protein export system and tightly synchronized *Plasmodium falciparum* cultures, we now show that export of parasite-encoded proteins across the parasitophorous vacuolar membrane is delayed, slower, and reduced in amount in hemoglobinopathic erythrocytes as compared to parasitized wild type red blood cells. Impaired protein export affects proteins targeted to the host cell cytoplasm, Maurer's clefts, and the host cell plasma membrane. Impaired protein export into the host cell compartment provides a mechanistic explanation for the reduced cytoadherence phenotype associated with parasitized hemoglobinopathic erythrocytes.

## INTRODUCTION

Malaria has plagued mankind since prehistoric times and will continue to do so in the foreseeable future as an effective vaccine is not yet available and resistance to currently used antimalarial chemotherapeutics is spreading. According to the latest estimates, malaria causes 225 million disease episodes and approximately 0.66 million deaths in the year 2012 (World Health Organization, 2013). The high death toll from malaria, particularly among young children, has placed a strong selective force on the human population, which, in turn, led to the emergence of polymorphisms in the human genome that protect carriers from severe malaria-related disease and death (Kwiatkowski, 2005; Taylor et al., 2013; Williams, 2006). The best established example of such a survival benefit is the sickle cell trait, an inheritable hemoglobin modification. Normal hemoglobin (HbA) consists of two α- and two β-globin chains. In sickle cell hemoglobin, the β-globin chain is altered at position 6 by a glutamate to valine substitution (Weatherall and Provan, 2000). Heterozygous carriers of the sickle cell hemoglobin (HbS) have a 10-fold lower risk of dying from malaria (Aidoo et al., 2002; Taylor et al., 2012; Williams, 2006; Williams et al., 2005). As a consequence, the sickle cell trait is highly prevalent in malaria endemic areas, particularly in Sub-Saharan Africa, with up to 25% of the native population carrying the allele despite the lethal consequences for homozygotes who frequently die of sickle cell disease at a young age (Nagel and Fleming, 1992; Piel et al., 2010; Simpore et al., 2002; Taylor et al., 2013). In addition to the sickle cell trait, there are several other hemoglobinopathies that confer a survival advantage in malaria infections. These include hemoglobin C (HbC), which, like HbS, contains an alteration at position 6 in the β-globin chain but instead of valine, glutamate is replaced by lysine, as well as α-thalassemia, a quantitative hemoglobinopathy where carriers produce reduced amounts of the α-globin chain, leading to an excess of unpaired β-globin in their red blood cells (Taylor et al., 2013; Williams, 2006).

Severe malaria is attributed to the intraerythrocytic life cycle of the protozoan parasite *Plasmodium falciparum* and the pathological cytoadhesive behavior of parasitized erythrocytes, which sequester in the deep vascular bed of inner organs (Beeson et al., 2002; Mackintosh et al., 2004; Miller et al., 2013; Silamut et al., 1999). By cytoadhering to the endothelial lining of venular capillaries, the parasite avoids splenic clearance mechanisms, but causes pathological sequelae in the affected blood vessel, such as diminished tissue perfusion, tissue hypoxia, and systemic microvascular inflammation (Mackintosh et al., 2004; Miller et al., 2013).

Hemoglobinopathies are thought to impact on the parasite's virulence through mechanisms that might include impaired intraerythrocytic development, reduced cytoadherence, and/or modulation of the host's immune responses, although the underpinning molecular processes are still under investigation and the relative contribution of the different models to protection remains to be established. For instance, several but not all studies have noted a reduced multiplication rate of *P. falciparum* in hemoglobinopathic erythrocytes (Friedman, 1978; Glushakova et al., 2014; Pasvol et al., 1978). LaMonte et al. (LaMonte et al., 2012) showed that certain microRNAs, miR-451 and miR-233, are enriched in sickle cell erythrocytes and can translocate into the parasite, where they inhibit mRNA translation and consequently affect the parasite growth (LaMonte et al., 2012). Studies conducted in a sickle cell murine model system have implicated accelerated turn-over of cytotoxic free heme in protection (Ferreira et al., 2011). Because of their variant hemoglobin, sickle cells release more heme into the plasma than do normal erythrocytes, which, in turn, stimulates the synthesis of hemoxygenase I by hematopoietic cells. Hemoxygenase I catalyzes the breakdown of heme, resulting in the production of the gasotransmitter carbon monoxide, which is thought to modulate the malaria-induced disease-mediating inflammatory reactions in the brain and other vital organs (Ferreira et al., 2011).

Recent evidence has pointed towards a role of hemoglobinopathies in interfering with cytoadhesion. Parasitized HbS, HbC and α-thalassemic erythrocytes display substantially reduced cytoadherence to human venular endothelial cells compared to infected wild type erythrocytes (Cholera et al., 2008; Fairhurst et al., 2005; Fairhurst et al., 2012; Krause et al., 2012; Taylor et al., 2012). The reduced capability to cytoadhere correlates with a number of other phenotype alterations. For effective cytoadhesion, the major adhesin molecule PfEMP1 needs to be placed in parasite-induced knob-like protrusions on the erythrocyte plasma membrane (Baruch et al., 1995; Goldberg and Cowman, 2010; Maier et al., 2009). Infected hemoglobinopathic erythrocytes, however, possess fewer and abnormally enlarged knobs (Cholera et al., 2008; Fairhurst et al., 2005; Fairhurst et al., 2003; Krause et al., 2012). Moreover, the amount of PfEMP1 molecules exposed on the cell surface is reduced, and the PfEMP1 molecules that are presented are aberrantly displayed (Cholera et al., 2008; Fairhurst et al., 2005; Fairhurst et al., 2003).

We have recently described further phenotypic anomalies in infected HbS and HbC erythrocyte, namely those that affect structural elements of the protein trafficking and sorting machinery that the parasite establishes within the host cell cytoplasm to direct proteins, such as PfEMP1, to the erythrocyte plasma membrane (Cyrklaff et al., 2011). For instance, Maurer's clefts, which serve as intermediary compartments for exported proteins and which usually form stacks of unilamellar membranes (Lanzer et al., 2006), have an amorphous appearance and their movements are aberrant in parasitized HbS and HbC erythrocytes (Cyrklaff et al., 2011; Kilian et al., 2013). Moreover, the parasite-induced actin filaments that normally connect the Maurer's clefts with knobs and which guide cargo vesicles from the Maurer's clefts to the erythrocyte surface are untypically short and unattached (Cyrklaff et al., 2011).

The finding that structural elements of the protein export system are aberrant in HbS and HbC erythrocytes begs the question of whether protein trafficking of parasite-encoded proteins to the host cell compartment is dysfunctional in hemoglobinopathic erythrocytes. If parasite proteins are not delivered to their destination at the right time and in the right amount, this might underpin the aberrant organization of the knobs, Maurer's clefts, the actin cytoskeleton, and eventually the impaired cytoadhesive behavior of parasitized hemoglobinopathic erythrocytes. To verify this model, we have studied the kinetics of protein trafficking from the parasite to different host cell compartments, including the cytoplasm, the Maurer's clefts and the plasma membrane. Our data show that protein export is delayed, slower, and with reduced amounts of exported protein in parasitized HbS and HbC erythrocytes.

## MATERIALS AND METHODS

### Ethical clearance

The study was approved by the ethical review boards of Heidelberg University and the Biomolecular Research Center (CERBA/Labiogene) at the University of Ouagadougou in Burkina Faso. Written informed consent was given by all blood donors.

### Red blood cells

The different hemoglobin genotypes were determined by polymerase chain reaction (PCR) restriction fragment length polymorphism (RFLP) and cellulose acetate electrophoresis as previously described (Modiano et al., 2001). The hemoglobinopathic erythrocytes were donated in Burkina Faso and immediately shipped at 4°C. After the arrival, the cells were washed 3 times with cold AB-transfection medium (RPMI 1640 medium supplemented with 2 mM l-glutamine, 25 mM Hepes, 100 µM hypoxanthine, 20 µg ml^−1^ gentamicin) and stored at 4°C. The cells used for infection experiments were not older than 3 weeks in total.

### Parasite strains

The *Plasmodium falciparum* strains 3D7 were used to monitor the conditional export of PfSBP1^CAD^ and SOL^CAD^. The GFP-tagged proteins PfSBP1^CAD^ and SOL^CAD^ have recently been described (Saridaki et al., 2008). To examine the presentation of PfEMP1 on the erythrocytic surface, the *P. falciparum* strain FCR3^CSA^, preselected for high capacity binding to CSA, was used (Scherf et al., 1998). The *var2csa* knockout strain FCR3^Δvar2CSA^, which exhibits a null CSA-binding phenotype, has been described (Viebig et al., 2005).

### Culture of *Plasmodium falciparum*

Hemoglobinopathic erythrocytes were infected with the MACS-purified (magnetic activated cell sorting, Miltenyi Biotech GmbH) late stages of *P. falciparum* (Sanchez et al., 2003). The eluted infected erythrocytes were washed twice in AB-transfection medium and used to inoculate a culture with a hematocrit of 5.0% of the appropriate red blood cells. The infected erythrocytes were maintained at 37°C under controlled atmospheric conditions (5% O_2_, 3% CO_2_, 96% humidity). The parasites containing PfSBP1^CAD^- and SOL^CAD^-plasmids were selected for using 5 nM WR99210. The parasitemia in these cultures was kept below 5% throughout. Cultures were synchronized using 5% d-sorbitol and/or gelatin flotation (Goodyer et al., 1994; Lambros and Vanderberg, 1979). The *P. falciparum* strain FCR3^CSA^ was selected for the adhesive phenotype every three weeks by adhesion to plastic flasks coated with 10 mg ml^−1^ CSA as previously described (Viebig et al., 2005).

### Induction of protein export and imaging

Trophozoite-stage parasites (18–22 h post invasion) were used to monitor the export of PfSBP1^CAD^ and SOL^CAD^. For the induction of the protein export, the anti-aggregation ligand AP21998, also known as D/D-Solubilizer (CloneTech), was used at a concentration of 1 µM as previously described (Saridaki et al., 2008). Cells were fixed in 4% paraformaldehyde and 0.0075% glutaraldehyde for 15 min in order to preserve the export phenotype of the early time points. These parasites were stored in phosphate buffered saline (PBS, pH 7.3) at 4°C prior to imaging. The infected erythrocytes were transferred into a chamber containing either physiological Ringer's solution (122.5 mM NaCl, 5.4 mM KCl, 1.2 mM CaCl_2_, 0.8 mM MgCl_2_, 11.0 mM d-glucose, 1.0 mM NaH_2_PO_4_ and 25.0 mM Hepes) supplemented with 5 nM WR99210 and 1 µM anti-aggregation ligand (live cells) or PBS (fixed cells). The protein export was examined using an LSM510 confocal laser scanning microscope (Carl Zeiss). The GFP in PfSBP1^CAD^ and SOL^CAD^ was excited at a wavelength of 488 nm with an argon laser (laser power 40%, transmission 3%, Plan-Apochromat 100×/1.4 oil DIC). The emission was captured with a 505 to 550 band pass filter. Images were analyzed using the Carl Zeiss AIM Image Examiner Version 4.2. Rates of protein export into the erythrocytic cytoplasm were determined using Fiji 1.45b 64-bit (http://Fiji.sc/Fiji). The deconvolution of the acquired images was performed with the software Huygens Essential 3.4 (Scientific Volume Imaging, SVI).

### Adhesion assay

The adhesion of *Plasmodium falciparum* FCR3^CSA^-infected erythrocytes to CSA was determined throughout the entire parasitic life cycle using static adhesion assay as previously described (Buffet et al., 1999; Viebig et al., 2005). Plastic petri dishes were prepared by pre-coating several areas of 20 mm^2^ overnight with 1 mg ml^−1^ purified CSA or 1% BSA as a control. Samples were taken at specified time points and 5×10^6^ infected erythrocytes were applied onto these spots for 1 h. After multiple washing steps, the adherent cells were fixed with 2% glutaraldehyde for at least 2 h. After staining for 10 min with 10% Giemsa, three randomly selected areas from each spot were imaged using Zeiss Axiovert 200M (objective 10×/0.25 air DIC).

### Flow cytometry

The presentation of total antigens was monitored using flow cytometry. Erythrocytes were taken from the culture at specific time points and fixed with 0.05% glutaraldehyde for 15 min. Afterwards, the cells were washed with 2% fetal calf serum in PBS. The cells were then labeled for 30 min with 3 µl of either pooled immune serum from 2 adult individuals living in a hyperendemic region in Burkina Faso or one uninfected adult as a control in a final volume of 50 µl PBS/fetal calf serum. After multiple washing steps, the erythrocytes were resuspended in 50 µl PBS/fetal calf serum containing 1∶100 fluorescein (FITC)-conjugated affinity-pure F(ab′)2 fragment donkey anti-human IgG (H+L), (Jackson ImmunoResearch Laboratories) and 1∶100 propidium iodide for 30 min. Uninfected erythrocytes were similarly treated in parallel as a control. After further washing steps, the fluorescence signal was measured. Briefly, the fluorescence displayed on the surface of 400 infected erythrocytes was determined using a FACScalibur (Becton Dickinson) and the CellQuest Pro Software 6.0.4 BD (Franklin Lakes) was used to further process and analyze the data.

### Scanning electron microscopy

Infected erythrocytes were deposited on a coverslip and fixed with 2.5% glutaraldehyde and 1% osmium tetroxide for 1 h. The cells were then dehydrated in aceton series (30–100%) and dried using critical point dryer. Alternatively, the dehydration was done by using ethanol series (30–100%) followed by soaking in 100% hexamethyldisalzan overnight. Dehydrated samples were mounted on studs and sputter-coated with 5–10 nm Au coat. The samples were viewed using a SEM Leo1530 at 10,000× magnification.

### Statistical analyses

The beginning of export and the export rate of PfSBP1^CAD^ and SOL^CAD^ were determined from the fluorescence exported to the erythrocytic cytoplasm by the parasite. The raw data were subsequently fitted using the sigmoid function:
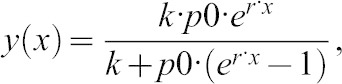
where *y* is the export in percent and *x* is the time in minutes. The parameters *k* (plateau value), *p*0 (intersection with *y*-axis) and *r* (a variable that decides the steepness of the ascent of the curve) were determined by fitting. With these three parameters, a high-resolution (2000 individual points) curve was calculated and numerically differentiated twice using the proper commands in the statistical program “R” (R Development Core Team, 2011). The x-value at which second derivative has its maximum was taken as onset of the protein export (Luu-The et al., 2005). To determine the export rate, the linear part of each sigmoid curve was extracted and fitted with a linear equation:
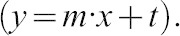
The slope (m) of this linear fit is the export rate in min^−1^. Error bars of the individual bars are the standard error of mean of the estimated slope as returned by “R”. Principal component analysis (Fig. 6) was performed using the appropriate commands in “R”. As input variables, we used the values for “export onset”, “export rate” and “amount of protein exported” compiled in Fig. 5. In addition, we calculated the Pearson correlation coefficient between each property according to:
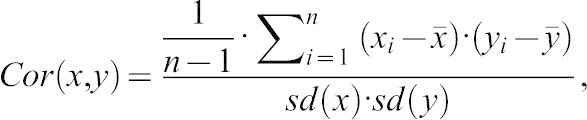
(*n*: number of individual data points, 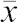: mean of x, 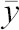: mean of y, sd(): standard deviation of data points). Statistical significance was subsequently determined using the Student's t-test. p-values <0.05 were considered significant.

## RESULTS

### Different kinetics of antigen presentation and cytoadherence in parasitized HbAS and HbCC erythrocytes

**Fig. 1.**
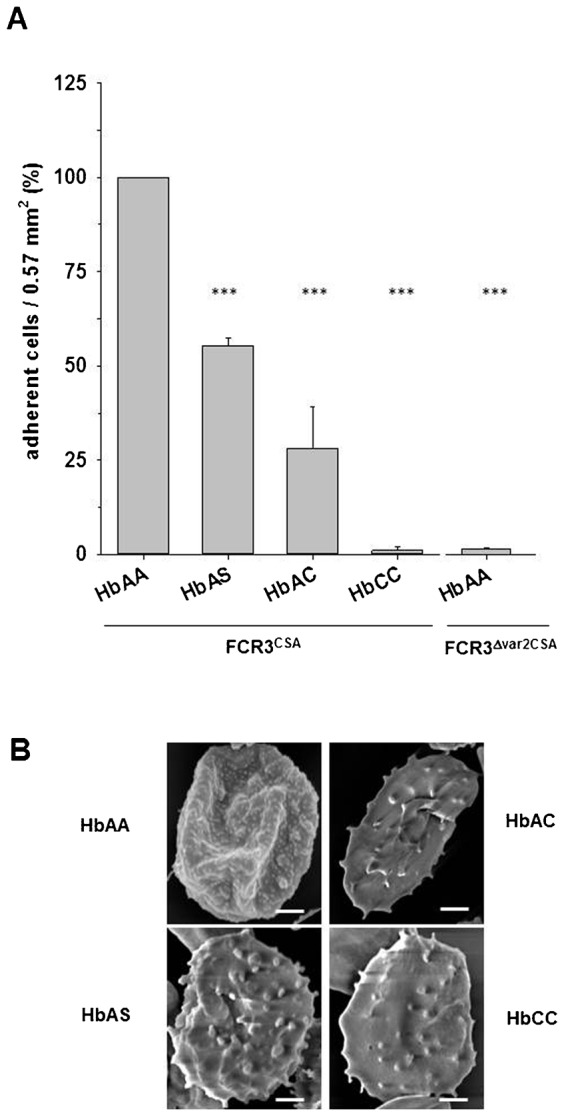
Adherence phenotype and knob morphologies exhibited by parasitized wild type and hemoglobinopathic erythrocytes. (A) The CSA-adherent *P. falciparum* strain FCR3^CSA^ was cultured in wild type erythrocytes (HbAA) and in erythrocytes containing the hemoglobin variants HbAS, HbAC, and HbCC. As a negative control, HbAA was infected with a *var2csa* knockout strain, termed FCR3^Δvar2CSA^ (Viebig et al., 2005), which exhibited a null CSA-binding phenotype. 5×10^6^ cells were investigated in each experiment. The number of adherent cells per field of view (0.57 mm^2^) were quantified and normalized to parasitized wild-type erythrocytes (HbAA). Statistics was performed using the Kruskal-Wallis test followed by a pairwise Wilcoxon rank-sum test with Bonferroni correction (***p<0.001). Error bars represent relative standard errors of the mean. (B) Morphology of parasitized HbAA, HbAS, HbAC, and HbCC erythrocytes, as imaged by scanning electron microscopy. Scale bars represent 2 µm.

We initially reproduced the previously described differential adhesion behavior of parasitized HbAA, HbAS, HbAC, and HbCC erythrocytes (Cholera et al., 2008; Fairhurst et al., 2003), using the *P. falciparum* strain FCR3^CSA^ (preselected for high capacity binding to bovine chondroitin sulfate A) and chondroitin sulfate A (CSA) as a widely used surrogate receptor for cytoadherence. In static binding assays performed concurrently, parasitized HbAS and HbAC erythrocytes showed a significant reduction (44% and 28%, respectively) in adherence compared to parasitized HbAA erythrocytes (Fig. 1A, p<0.001). Parasitized HbCC erythrocytes showed little to no cytoadherence. This was comparable to the null CSA-binding phenotype displayed by FCR3^Δvar2CSA^ (Viebig et al., 2005), where the *var2CSA* gene that exclusively confers binding to CSA was disrupted (Fig. 1A). Reduced cytoadherence correlated with altered knob morphology and density; parasitized HbAS, HbAC, and HbCC erythrocytes had fewer and abnormally enlarged knobs, compared to infected wild type erythrocytes, as determined by scanning electron microscopy (Fig. 1B). This was consistent with previous reports (Cholera et al., 2008; Cyrklaff et al., 2011; Fairhurst et al., 2003).

**Fig. 2.**
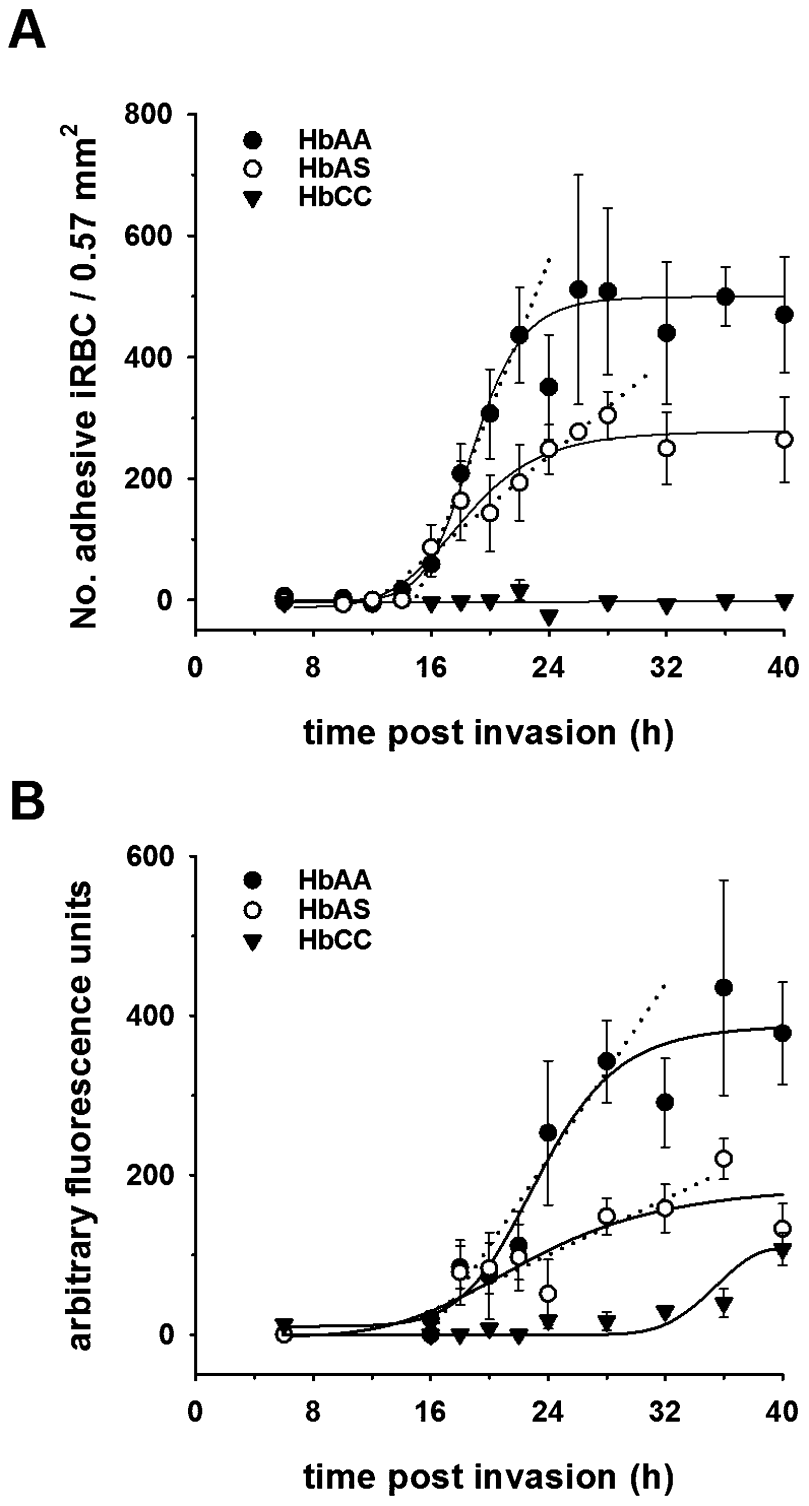
CSA-Adhesion and surface antigen presentation during the intraerythrocytic life cycle of *P. falciparum* in infected wild type and hemoglobinopathic erythrocytes. (A) Parallel adhesion assays were performed on tightly synchronized FCR3^CSA^ at specified time points (6–40 h post invasion) during the intraerythrocytic life cycle. 5×10^6^ cells were investigated in each experiment. The number of CSA-adhesive parasitized erythrocytes per field of view (0.57 mm^2^) was quantified by image analysis. Each data point is an average of at least three independent experiments±standard error of mean. Sigmoidal functions were fitted to each data set and the kinetics of adherence was quantified by fitting linear functions to the rising sections of each data set. (B) The temporal appearance of total antigens on the surface of parasitized HbAA, HbAS, and HbCC erythrocytes during the intraerythrocytic life cycle, as quantified by flow cytometry. Pooled sera from residents of a malaria holoendemic region in Burkina Faso were used for detection of parasitic surface antigens. Each data point is an average of at least three independent experiments±standard error of mean. The slopes of the linear parts of the data points were fitted to linear functions to quantify the kinetics of surface antigen presentation.

Next, we investigated the time course of adherence to CSA by determining the amount of adherent cells throughout the intraerythrocytic life cycle of *P. falciparum*, with samples taken in time intervals of 2 to 4 h, using tightly synchronized cells. In parasitized HbAA erythrocyte, the adherence phenotype appeared 16 h post invasion and then increased until 24 h post invasion before reaching a plateau value (Fig. 2A), consistent with previous reports (Gardner et al., 1996; Kriek et al., 2003). Time courses of parasitized HbAS erythrocytes, which were performed in parallel with infected wild type red blood cells, revealed a significantly slower temporal increase in the number of adherent cells and a lower final plateau level (Fig. 2A; p<0.01). Fitting linear functions to the rising sections of the data points provided a quantitative readout, in terms of the slopes, for the differential adherence kinetics displayed by parasitized HbAA and HbAS erythrocytes. In parasitized HbAA erythrocytes, adherence rose with a slope of 61±4 cells h^−1^, as compared to 20±2 cells h^−1^ for parasitized HbAS erythrocytes (p<0.01 in Fig. 2A). Parasitized HbCC erythrocytes did not show any significant binding to CSA during the intraerythrocytic life cycle (Fig. 2A).

We determined, in paired and parallel assays, the temporal appearance of total antigens on the surface of HbAA, HbAS and HbCC erythrocytes infected with FCR3^CSA^ during the intraerythrocytic life cycle, using pooled sera from residents of a malaria holoendemic region in Burkina Faso. The FACS analysis revealed clear differences in the time courses of antigen presentation between the different parasitized red blood cells. In HbAA infected erythrocytes, the first surface antigens were detected 16 h post infection. The amount of presented antigens then rose with a slope of 30±4 arbitrary fluorescence units h^−1^ before it reached a plateau level 30 h post invasion (Fig. 2B). In parasitized HbAS erythrocytes, antigen presentation rose with a significantly flatter slope of 10±4 arbitrary fluorescence units h^−1^ (p<0.01). Moreover, the final plateau value was only half that of infected wild type erythrocytes (Fig. 2B). Even more pronounced were the differences in parasitized HbCC erythrocytes. Antigens were not detected on the cell surface before 36 h post invasion and the total amount of antigen presented was even lower than that seen in parasitized HbAS erythrocytes (Fig. 2B). These data indicate major deviations in the timing and the amount of cytoadherence and antigen presentation in parasitized HbAS and HbCC erythrocytes, relative to the wild type controls.

### Aberrant protein trafficking across the parasitophorous vacuolar membrane

We have recently described a conditional protein export system in *P. falciparum* based on the conditional aggregation domain (CAD) that allows protein trafficking to destinations within the host erythrocyte to be controlled (Saridaki et al., 2008). Proteins of interest fused to the CAD domain self-aggregate in the parasite's ER in a reversible manner and only continue their trafficking path upon the addition of a small membrane-permeable ligand. We used this conditional export system to further analyze potential differences in protein trafficking associated with erythrocytes containing HbS and HbC. Two proteins were studied: (i) an artificial, soluble, PEXEL-containing protein (consisting of the first 80 amino acids of a STEVOR protein), termed SOL^CAD^, destined for export into the cytoplasm of the host cell (Saridaki et al., 2008); and (ii) a membrane bound non-PEXEL PfSBP1 fusion protein, termed PfSBP1^CAD^, targeted to Maurer's clefts (Saridaki et al., 2008). Both proteins were tagged with the green fluorescence protein to follow their trafficking. The corresponding genes were episomally expressed in the *P. falciparum* strain 3D7, which was continuously cultured in vitro using the following erythrocyte variants: HbAA, HbAC, HbAS, HbCC, and HbSC.

**Fig. 3.**
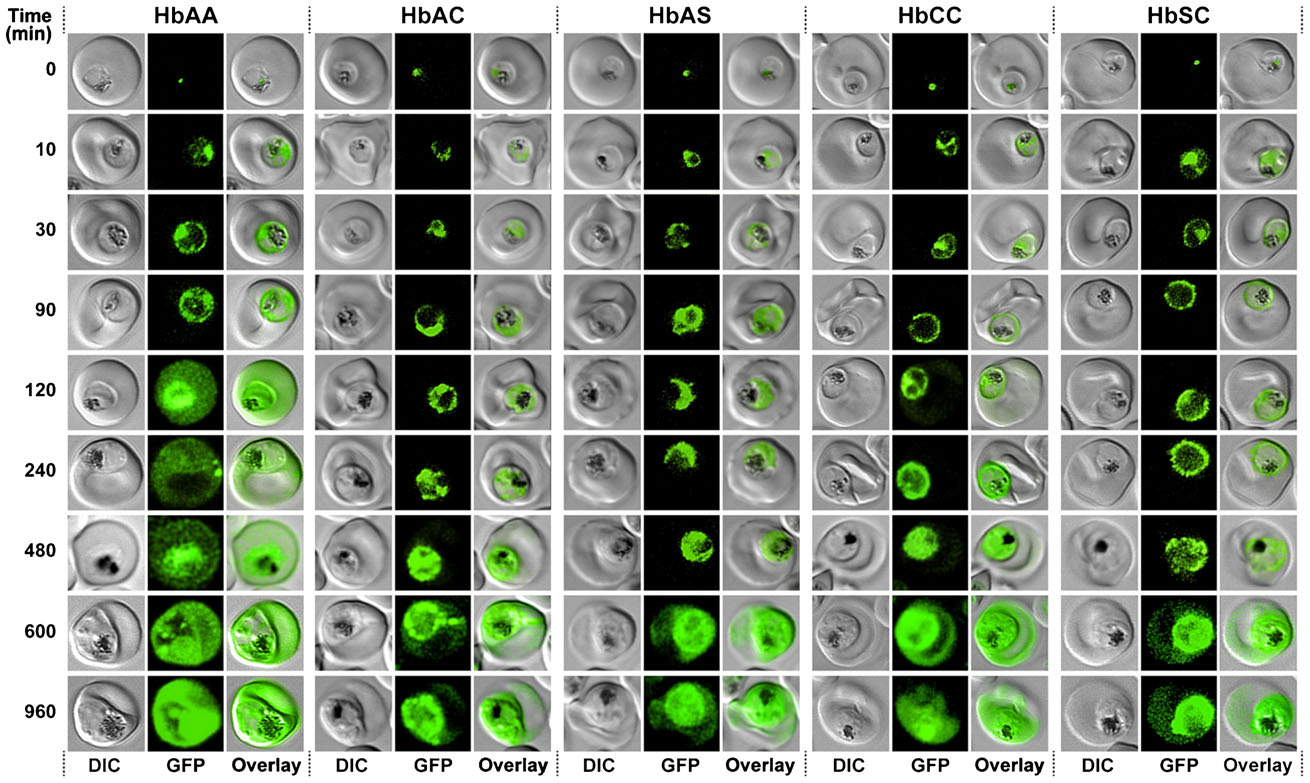
Kinetics of export of SOL^CAD^ in various *P. falciparum*-infected erythrocytes. *P. falciparum*-infected erythrocytes were taken from culture at the time points indicated after the addition of the anti-aggregation ligand and immediately analyzed by live cell confocal fluorescence microscopy. Tightly synchronized cultures at the trophozoite-stage (18–22 h post invasion) were used. Images shown are representative examples for the export phenotypes that were used for later quantification.

In the absence of the anti-aggregation ligand, both SOL^CAD^ and PfSBP1^CAD^ were retained in the parasite's ER, consistent with previous reports (Saridaki et al., 2008). To study the kinetics of protein export, the anti-aggregation ligand was added to highly synchronized parasite cultures at the early trophozoite stage (18–22 h post invasion) and the time courses of protein export to the erythrocyte cytosol and the Maurer's clefts, respectively, were monitored over the next 960 min. In the case of SOL^CAD^, fluorescence spread from its initial focus in the parasite's ER to the parasitophorous vacuolar lumen within 90 min following the addition of the anti-aggregation ligand (Fig. 3). No major differences in intra-parasitic protein trafficking were apparent between wild type and hemoglobinopathic erythrocytes at this stage. After 120 min, a homogenous fluorescence signal was detected in the erythrocyte cytosol but only in parasitized HbAA erythrocytes and not in erythrocytes containing hemoglobin variants. In parasitized HbAC, HbAS, HbCC, and HbSC erythrocytes, SOL^CAD^ remained in the parasitophorous vacuole, and it was not until the 600 min time point that a clear fluorescence signal was detected in the host cell cytoplasm.

**Fig. 4.**
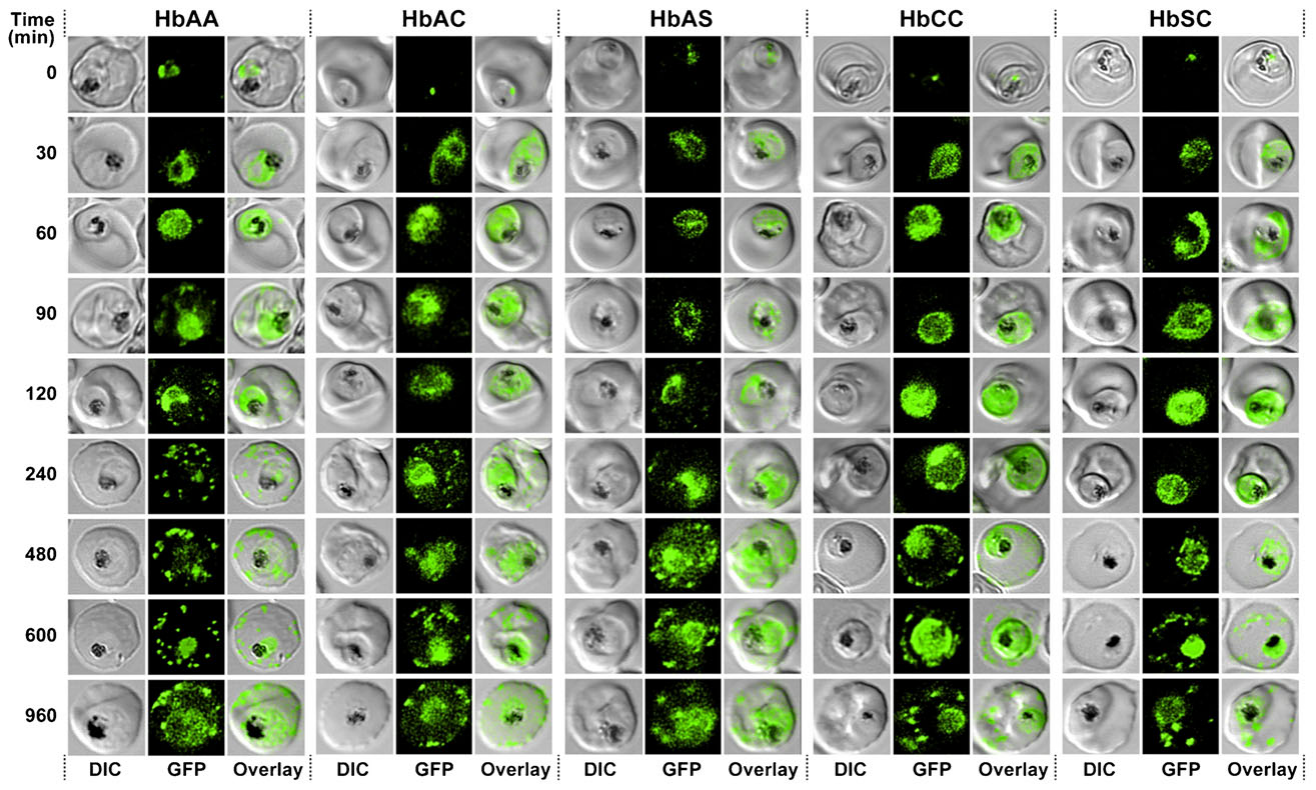
Kinetics of export of PfSBP1^CAD^ in various *P. falciparum*-infected erythrocytes. *P. falciparum*-infected erythrocytes were taken from culture at the time points indicated after the addition of the anti-aggregation ligand and immediately analyzed by live cell confocal fluorescence microscopy. Tightly synchronized cultures at the trophozoite-stage (18–22 h post invasion) were used. Images shown are representative examples for the export phenotypes that were used for later quantification.

Similar results were obtained for PfSBP1^CAD^. Again, the protein was trafficked from the parasite's ER to the parasite's periphery within 60 min upon addition of the anti-aggregation ligand, with no apparent differences in the time courses between wild type and variant erythrocytes. Between 60 to 90 min upon the addition of the anti-aggregation ligand PfSP1^CAD^ had reached the Maurer's clefts, but only in parasitized wild type erythrocytes. In parasitized erythrocytes containing the hemoglobin variants S and C, export beyond the parasitophorous vacuolar membrane was substantially delayed (Fig. 4).

**Fig. 5.**
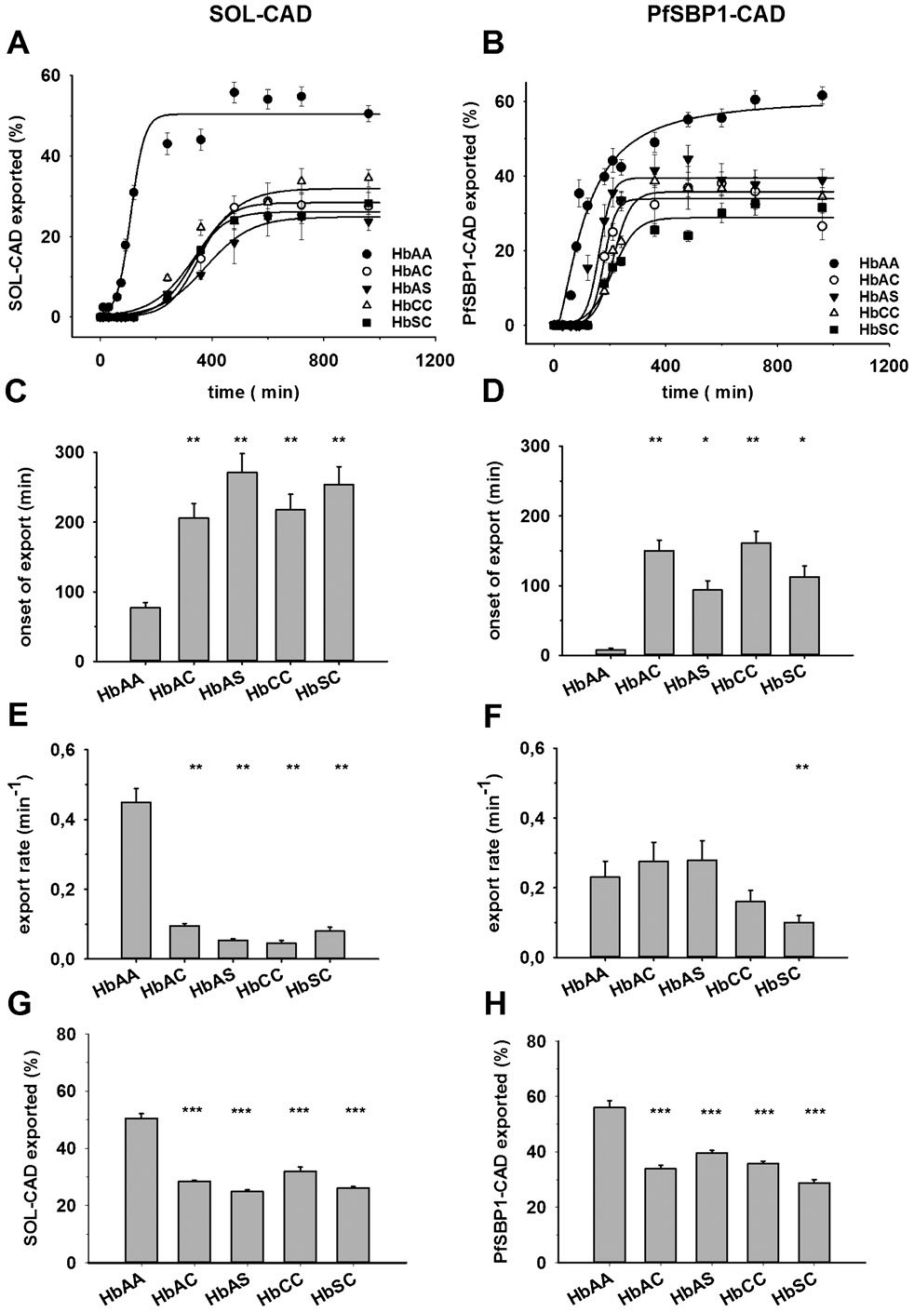
Quantification and export kinetics of SOL^CAD^ and PfSBP1^CAD^ in various parasitized erythrocytes. (A,B) Time courses of conditional SOL^CAD^ or PfSBP1^CAD^ export following the addition of the anti-aggregation ligand. The figure shows the relative amount of SOL^CAD^ (A) and PfSBP1^CAD^ (B), normalized to total cellular fluorescence, exported from the parasite over a period of 960 min following induction, as quantified by fluorescence intensity from microscopic images (see Figs 3 and 4). A logistic curve was fitted to each data set. (C,D) The onset of export was calculated from the fitted curves using the second derivative maximum method (Luu-The et al., 2005) and plotted for each erythrocyte variant. Statistical significance was tested using one-way ANOVA followed by a pairwise t-test with Bonferroni adjustment. *p<0.05; **p<0.01. (E,F) The rate constant of export was determined by fitting a straight line to the linear part of the sigmoid curves of panels A and B. The error bars represent the standard error of mean of the slope estimate of the regression line. Statistical testing was again carried out by one-way ANOVA followed by a pairwise t-test with Bonferroni adjustment. **p<0.01. (G,H) Plateau values of export were obtained from the initial curve fits and analyzed as a function of the different erythrocytes. Statistical significance was determined by one-way ANOVA followed by a pairwise t-test with Bonferroni adjustment. **p<0.001. Error bars correspond to the standard error of the means.

To better assess the kinetics of protein export in the different parasitized erythrocytes, we quantified the data by determining the fraction of fluorescence that was present in the erythrocyte compartment (in reference to the total cellular fluorescence) in at least 40 cells, obtained from three independent biological replicates, for each time point and parasitized erythrocyte variant. Logistic functions were then fitted to the data points, which yielded the plateau value of protein export plotted in Fig. 5A,B for SOL^CAD^ and PfSBP1^CAD^, respectively. The onset of export across the PVM was subsequently determined, using the second derivative maximum method (Luu-The et al., 2005), and the export rate was calculated by fitting a linear function to the linear part of each sigmoidal curve.

As shown in Fig. 5C, export of SOL^CAD^ into the host cell cytosol commenced around 80±10 min after the addition of the anti-aggregation ligand. Export then continued with a rate of 0.45±0.04 min^−1^ until a maximal plateau value of 50±5% (in reference to the total cellular fluorescence) was reached (Fig. 5E,G). Significantly different parameters were obtained for parasitized hemoglobinopathic erythrocytes: Onsets of export were delayed by 120 to 190 min (Fig. 5C); the export rates were slower (0.05 to 0.1 min^−1^) (Fig. 5E); and the final export plateau values were lower (24 to 31%), compared to parasitized wild type erythrocytes. The variations between the different parasitized mutant erythrocytes were not statistically significant.

Comparable results, but with some distinctions, were obtained for PfSBP1^CAD^ (Fig. 5B). Again the onsets of export, in this case to the Maurer's clefts, were delayed when the parasites were cultured in hemoglobinopathic erythrocytes as compared to wild type red blood cells (95 to 160 min and 10 min, respectively; p<0.01 in Fig. 5D). Once export started, parasitized HbAC, HbAS, and HbCC erythrocytes progressed at a rate comparable to that observed in parasitized HbAA erythrocytes, or at a slower rate as observed for parasitized HbSC erythrocytes (0.23±0.05 min^−1^ for HbAA, 0.28±0.06 min^−1^ for HbAC and HbAS, 0.16±0.03 min^−1^ for HbCC and 0.10±0.02 min^−1^ for HbSC). Approximately 300 min after the addition of the anti-aggregation ligand, the amount of PfSBP1^CAD^ associated with the Maurer's clefts reached a plateau level. This final level of protein export was consistently lower in parasitized erythrocytes with altered hemoglobin compared to parasitized HbAA erythrocytes (30 to 40% and 56%, respectively; p<0.001, Fig. 5H). The discrepancies in export onset between the sample images shown in Figs 3 and 4 and the values shown in Fig. 5 are due to higher sensitivity of the image quantification software in comparison to the human eye, both on screen and in print.

**Fig. 6.**
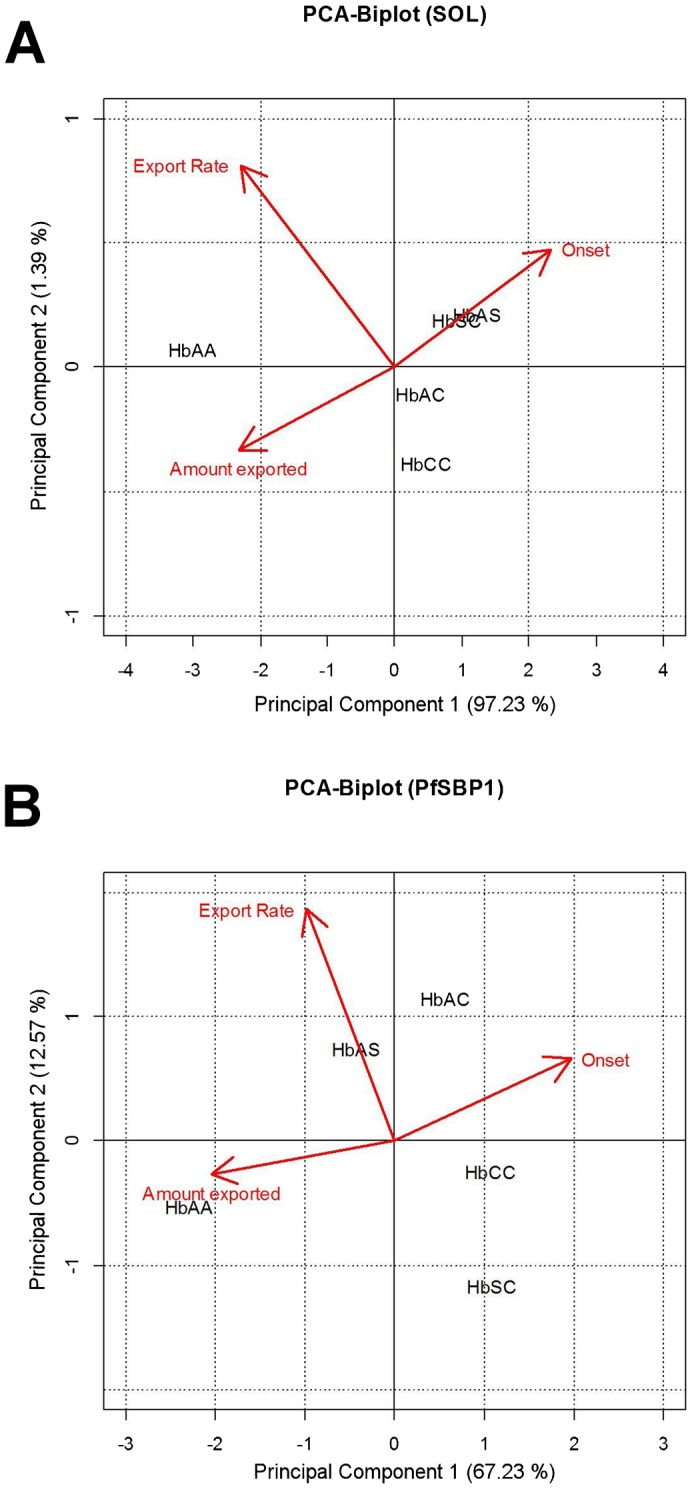
Principal component analysis (PCA) of SOL^CAD^ and PfSBP1^CAD^ export kinetics in erythrocytes containing different hemoglobin variants. The PCA for SOL^CAD^ (A) and PfSBP1^CAD^ (B) in various hemoglobinopathic erythrocytes were calculated using the export onset, rate of export, and amount of protein exported (relative to total cellular fluorescence). The biplot of the first two principal components is shown and the percentage of the total variance explained by each principal component is included in parenthesis. The variance is taken as a measure for information content for the particular axis. The plot is overlaid with the eigenvectors (red arrows) whose length and direction indicate how influential a variable is (Abdi and Williams, 2010). In both biplots, the principal component 1 was primarily driven by the amount of protein exported and secondarily by the onset of protein export. The principal component 2 was predominantly associated with the protein export rate. Clustering of different hemoglobin variants indicate similarities in the protein export behaviors of these erythrocytes.

Using principal component analysis (Abdi and Williams, 2010), we further investigated the possibility of underlying patterns within the export parameters, i.e., onset, rate, and amount of exported protein (Fig. 6). A principal component analysis reduces high-dimensional datasets in order to map them onto plots with fewer dimensions, while maintaining as much information as possible. The variance is taken as a measure of information content for the particular axis. The three properties investigated here (translating into three dimensions) could be successfully projected onto a two dimensional space. The datasets could be described almost entirely by only the first two principal components, covering an explained variance of 98% for SOL and 80% for PfSBP1. The length of the eigenvectors (red arrows) and the directions in which they point indicate how influential a variable is and how different variables are correlated.

It is evident from Fig. 6A,B that the amount of protein export and the onset of protein export are negatively correlated with each other, i.e., delayed protein export is associated with low amounts of exported protein (the Pearson correlation coefficients and the corresponding p-values are: −0.98 and <0.01 for SOL^CAD^, and −0.89 and 0.04 for PfSBP1^CAD^). Both onset and amount of protein export largely define the principal component 1. The protein export rate, which primarily drives the principal component 2, revealed protein dependent correlation characteristics. In the case of SOL^CAD^, the export rate correlated negatively with the onset of protein export and positively with the amount of protein export (The Pearson correlation coefficients and the corresponding p values are: −0.95 and 0.015, and 0.95 and 0.012, respectively), whereas in the case of PfSBP1^CAD^ the export rate was independent of the two other variants (−0.17 and >0.5, and 0.33 and >0.5, respectively). The principal component analysis further revealed similarities in the protein export behaviors of the different parasitized erythrocytes.

For SOL^CAD^, two clusters can be observed. The first cluster consists of hemoglobinopathic erythrocytes carrying HbS, i.e., HbAS and HbSC erythrocytes, and is characterized by delayed onset and reduced amount of protein export (Fig. 6A). The second group is formed by erythrocytes carrying HbAC and HbCC and has lower export rates and slightly increased (though not statistically significant) amounts of protein export than the first group (Fig. 6A). Apparently, HbS has a strong dominating effect over HbA and HbC, in that it severely impacts on onset and amount of protein export into the host cell compartment. HbAA wild type can be clearly distinguished from all the hemoglobinopathic erythrocytes (Fig. 6A).

The principal component analysis of PfSBP1^CAD^ revealed a similar pattern; again, the HbAA wild type is clearly separated from all variant hemoglobins (Fig. 6B). However, a slightly different clustering pattern is observed amongst the hemoglobinopathies. Here, the heterozygous HbA erythrocytes are grouped together, indicating that the effects of the remaining HbA are the deciding factor for the export kinetics of the membrane-bound PfSBP1^CAD^. The absence of HbA, such as in HbCC and HbSC erythrocytes, creates a different cluster, which shows a strong impairment in PfSBP1^CAD^ export kinetics (Fig. 6B).

## DISCUSSION

Here we explored the hypothesis that hemoglobin S and C affect trafficking of exported parasite-encoded proteins within the host cell compartment. The study was inspired by our recent finding that structural constituents of the protein trafficking and sorting machinery are malformed in parasitized HbCC and HbSC erythrocytes (Cyrklaff et al., 2012; Cyrklaff et al., 2011). We found that the kinetics of protein export was anomalous when parasites were grown in hemoglobinopathic erythrocytes, as exemplified for surface antigens, the Maurer's clefts-associated membrane protein PfSBP1, and an artificial soluble protein targeted into the host cell cytoplasm.

Aberrant protein export is consistent with most models posited to explain the protective effect of hemoglobinopathic erythrocytes on severe malaria. *P. falciparum* exports a few hundred proteins into the host erythrocyte compartment (Goldberg and Cowman, 2010; Heiber et al., 2013; Maier et al., 2009; van Ooij et al., 2008). If these proteins are delivered at the wrong moment in time or in insufficient quantity then this is likely to impact on physiological and pathophysiological functions of the parasite. As a consequence, intraerythrocytic development of the parasite might be impaired, particularly if parasite-encoded transporters and channels are affected that create permeation pathways for nutrient uptake and ion homeostasis across the host erythrocyte cytoplasm (Nguitragool et al., 2011). Indeed several studies have reported lower intraerythrocytic multiplication rates of *P. falciparum* in HbS and HbC containing erythrocytes, although other studies failed to confirm this result (Fairhurst et al., 2003; Friedman et al., 1979; LaMonte et al., 2012; Olson and Nagel, 1986; Pasvol, 1980; Pasvol et al., 1978) and a meta-analysis revealed that malaria patients harboring hemoglobinopathies can have parasitemias as high as those seen in patients with normal hemoglobin (Taylor et al., 2013; Taylor et al., 2012). Our own data suggest that the parasite develops normally in the different red blood cell variants under continuous in vitro culture, although there is a statistically insignificant trend of a slightly lower replication rate when parasites are grown in hemoglobinopathic erythrocytes (Kilian et al., 2013). Similarly, delayed and insufficient export of the knob-associated histidine-rich protein, the major constituent of the knobs, and PfEMP1 into the host compartment may, at least in part, explain why parasitized hemoglobinopathic red blood cells possess fewer and abnormally enlarged knobs, why they present less PfEMP1 on the surface, and why their ability to cytoadhere is diminished (Cholera et al., 2008; Fairhurst et al., 2005; Fairhurst et al., 2012) (Fig. 2A).

Cryotomographic images have recently shown that structural features of the protein trafficking and sorting system established by the parasite in the host cell cytoplasm are malformed in parasitized HbCC and HbSC erythrocytes (Cyrklaff et al., 2011). For instance, the Maurer's clefts, serving as intermediary compartments for proteins en route to the plasma membrane, have an amorphous appearance (Cyrklaff et al., 2011), which is quite distinct from the typical stacked unilamellar membrane profiles (Lanzer et al., 2006). In addition, host actin reorganization progresses only marginally in parasitized HbCC and HbSC erythrocytes. Usually the parasite mines the actin of the erythrocyte membrane skeleton to generate a network of long filaments that connect the Maurer's clefts with the knobs and which seem to direct cargo vesicles towards the erythrocyte plasma membrane (Cyrklaff et al., 2012; Cyrklaff et al., 2011). The parasite-induced actin filaments are much shorter in parasitized HbCC and HbSC erythrocytes and they do not link the Maurer's clefts with the host cell plasma membrane (Cyrklaff et al., 2012; Cyrklaff et al., 2011). Although tomographic images are not yet available for parasitized HbAS and HbAC erythrocytes, it is conceivable that Maurer's clefts morphology and host actin reorganization are also affected in these hemoglobinopathic cells.

The degree of functional impairment seems to vary among the haemoglobinopathies, as suggested by principal components analyses of the three export parameters: onset of export, rate of export, and amount of protein exported, which were recorded for each of the different erythrocytes. In the case of SOL^CAD^, parasitized HbAS and HbSC erythrocytes and parasitized HbAC and HbCC erythrocytes form a similarity cluster each. In the case of the Maurer's clefts associated protein PfSBP1^CAD^, parasitized HbAS and HbAC erythrocytes cluster and parasitized HbCC and HbSC erythrocytes cluster. These differences in groupings might be explained by the different classes of exported protein that SOL^CAD^ and PfSBP1^CAD^ represent. SOL^CAD^ is a soluble PEXEL containing protein (Marti et al., 2004) and PfSBP1^CAD^ is a membrane-associated PEXEL-negative exported protein (Heiber et al., 2013; Saridaki et al., 2009). Although both PEXEL and PEXEL-negative exported proteins share many functional and structural features along their trafficking pathway, including unfolding and translocation across the parasitophorous vacuolar membrane via a common translocon (Beck et al., 2014; Elsworth et al., 2014; Heiber et al., 2013), there may be slight differences in protein handling and processing that are affected by the various hemoglobinopathies to different degrees. Irrespectively, the principal component analysis of SOL^CAD^ and PfSBP1^CAD^ revealed some common principles: Firstly, a late onset of export is generally highly correlated with a low amount of protein export. Secondly, the export rate is correlated with the onset of export or the amount of protein export only for SOL^CAD^ but not for PfSBP1^CAD^. This finding might again point towards some differences in the protein export pathways between a PEXEL and a PEXEL-negative protein. Thirdly, HbCC seems to be the most effective hemoglobin variant when it comes to affecting protein export kinetics of both SOL^CAD^ and PfSBP1^CAD^. The highly impaired protein export displayed by HbCC erythrocytes correlates well with the almost null adhesion phenotype of these cells (compare Fig. 6A,B with Fig. 1A). HbSC erythrocytes displayed an even more impaired protein export phenotype than did HbCC erythrocytes but only for PfSBP1^CAD^ not for SOL^CAD^.

While malfunctioning protein trafficking from the Maurer's clefts to the erythrocyte plasma membrane provides a plausible explanation for diminished total antigen presentation and reduced cytoadhesion, the results obtained using the conditional protein trafficking system suggest a more nuanced model. Unexpectedly, trafficking of fluorescently labeled PfSBP1^CAD^ and SOL^CAD^ was delayed at the parasitophorous vacuolar membrane. This membrane, which separates the parasite from the host cell, is a highly selective barrier that lets only those parasite-encoded proteins pass via a translocon that are allotted for export into the host cell compartment (Beck et al., 2014; Elsworth et al., 2014). Why proteins are held up in the parasitophorous vacuolar lumen is unclear, but might involve a translocation process that is malfunctioning in hemoglobinpopathic erythrocytes.

HbS and HbC containing erythrocytes are characterized by a redox imbalance due to the instability of their hemoglobin (Chaves et al., 2008; Darghouth et al., 2011). Unlike normal hemoglobin, HbS and HbC are prone to oxidation, resulting in increased amounts of irreversible hemichromes, free heme, and free iron, which themselves act as oxidants (Bauminger et al., 1979; Chaves et al., 2008; Hebbel, 1991). For instance, ferryl hemoglobin can oxidize actin, which was shown to reduce actin polymerization rates and affect actin dynamics (Abraham et al., 2002; Cyrklaff et al., 2011; Farah et al., 2011; Jarolim et al., 1990). On the basis of these considerations, we have recently proposed that the redox imbalance triggered by the instability of HbS and HbC interferes with host actin reorganization and subsequently with the organization of Maurer's clefts in hemoglobinopathic erythrocytes (Cyrklaff et al., 2012; Cyrklaff et al., 2011). Whether components of the translocon or factors assisting translocation are affected by the increased oxidative state present in hemoglobinopathic erythrocytes remains to be seen. Interestingly, several membrane protein supercomplexes recruit actin to stabilize their assembly (Abrami et al., 2010). This includes the ribosome-translocon complex of the endoplasmic reticulum which, after binding of the palmitoylated chaperone calnexin, binds to the actin cytoskeleton (Lakkaraju et al., 2012). It is tempting to speculate that the impaired protein translocation across the parasitophorous vacuolar membrane relates not only to a malfunctioning translocon but also to the inability of the translocon to recruit actin in hemoglobinopathic erythrocytes.
